# CXCR4/CXCL12 Participate in Extravasation of Metastasizing Breast Cancer Cells within the Liver in a Rat Model

**DOI:** 10.1371/journal.pone.0030046

**Published:** 2012-01-13

**Authors:** Claudia Wendel, André Hemping-Bovenkerk, Julia Krasnyanska, Sören Torge Mees, Marina Kochetkova, Sandra Stoeppeler, Jörg Haier

**Affiliations:** 1 Department of General and Visceral Surgery, University Hospital Muenster, Muenster, Germany; 2 Chemokine Biology Division, School of Molecular and Biomedical Science, University of Adelaide, Adelaide, Australia; 3 Comprehensive Cancer Center Muenster, University Hospital Muenster, Muenster, Germany; Health Canada, Canada

## Abstract

**Introduction:**

Organ-specific composition of extracellular matrix proteins (ECM) is a determinant of metastatic host organ involvement. The chemokine CXCL12 and its receptor CXCR4 play important roles in the colonization of human breast cancer cells to their metastatic target organs. In this study, we investigated the effects of chemokine stimulation on adhesion and migration of different human breast cancer cell lines in vivo and in vitro with particular focus on the liver as a major metastatic site in breast cancer.

**Methods:**

Time lapse microscopy, in vitro adhesion and migration assays were performed under CXCL12 stimulation. Activation of small GTPases showed chemokine receptor signalling dependence from ECM components. The initial events of hepatic colonisation of MDA-MB-231 and MDA-MB-468 cells were investigated by intravital microscopy of the liver in a rat model and under shRNA inhibition of CXCR4.

**Results:**

In vitro, stimulation with CXCL12 induced increased chemotactic cell motility (p<0.05). This effect was dependent on adhesive substrates (type I collagen, fibronectin and laminin) and induced different responses in small GTPases, such as RhoA and Rac-1 activation, and changes in cell morphology. In addition, binding to various ECM components caused redistribution of chemokine receptors at tumour cell surfaces. In vivo, blocking CXCR4 decreased extravasation of highly metastatic MDA-MB-231 cells (p<0.05), but initial cell adhesion within the liver sinusoids was not affected. In contrast, the less metastatic MDA-MB-468 cells showed reduced cell adhesion but similar migration within the hepatic microcirculation. Conclusion: Chemokine-induced extravasation of breast cancer cells along specific ECM components appears to be an important regulator but not a rate-limiting factor of their metastatic organ colonization.

## Introduction

Metastasis is the result of multiple sequential steps and is a highly organized, non-random, and organ-selective process [1]. Tumour cell interactions with endothelium and subendothelial extracellular matrix (ECM) constitute crucial factors in determining the organ preference of metastasis. The interplay between malignant tumour cells and their surrounding ECM has been implicated at nearly every stage of the metastatic process; ranging from steps that involve the local invasion of tumour cells away from the primary tumour to those that are involved in mediating extravasation through microvessel-associated basement membranes at the site(s) of metastasis formation [2]. Initial arrest and attachment of circulating tumour cells in the secondary organs are believed to be crucial events for haematogenous metastasis, but the actual processes in in vivo conditions remain a matter of debate [3], [4], [5], [6], [7]. Adhesion of circulating tumour cells to microvascular endothelial cells and their underlying ECM represents an initial event of metastatic organ colonisation alongside extravasation into the host organ parenchyma [8].

Many of these characteristics for metastasis formation are related to tumour cell adhesion and migration with haptotactic guidance. Chemotactic molecules, such as chemokines and their receptors, were also shown to play an important role in organ-specific colonization of metastatic tumour cells [9], [10], [11]. Physiologically, chemokines are active on neutrophils and T-lymphocytes (-CXC- type), while –CC- type chemokines are active on monocytes and lymphocytes, predominantly mediating stimulation of leukocyte chemotaxis during inflammation [9]. Tumour cell migration and metastasis appear to share many similarities with leukocyte trafficking. Müller et al. [12] reported that tumour cells express a distinct pattern of functionally active chemokine receptors which correlates with their metastatic behaviour.

Breast cancer is an example for a tumour with an organ-specific pattern of distant metastasis formation. It mainly colonizes lung, liver, lymph nodes and bone marrow, all of which are abundant sources of chemokine ligands [12], [13]. Overexpression of chemokines - especially of CXCR4 and CCR7 - was observed in breast cancer cells and surgical specimens, but chemokine receptors are also highly expressed in other tumour types including cancers of epithelial, mesenchymal and hematopoietic origin [14]. The role of CXCR4 in the metastatic cascade of breast cancer and also its ability to predictpatient survival have been intensively studied [15]. Several groups found that CXCR4 and its ligand CXCL12 can promote tumour cell migration and invasion [9], [12], [16], [17], [18], [19], [20]. For example, CXCL12/SDF-1α induced cellular responses, such as calcium mobilization, actin polymerization, and chemotaxis in metastatic cells, whereas non-invasive cells were unresponsive [21]. In addition, CXCL12 activated multiple signalling pathways downstream of G-proteins in highly invasive cells but failed to activate downstream kinase cascades in non-invasive cell lines [21]. Since chemotactic tumour cell characteristics are related to cellular interactions with ECM components, the composition of these matrix proteins appears to be relevant to the metastatic process [22]. AsECM composition differs between organs and tissue types, chemokine activity in breast cancer cells may be dependent on ECMavailability, as is true for haematogenous cells. In T-lymphocytes, for example, chemokines presented within a collagen matrix increased depth of migration of infiltrating cells in vitro whereas the presence of fibronectin within the collagen substrate modulated their adhesive and migratory properties [23].

Recently, we reported that the hepatic subendothelial space of Dissé is constituted of fibronectin (FN), von Willebrandt-factor, type I (C I) and type IV collagen (C IV) and small amounts of laminin-5 (LN). Using different types of inhibitors, we found that initial adhesion of circulating tumour cells within the liver sinusoids was mainly mediated by RGD-dependent integrin binding to FN. In contrast, binding to C I or C IV was not involved in initial cell adhesion but C I enabled tumour cell extravasation into the liver parenchyma via α2β1-integrins. LN was not involved in any of these steps [24]. Taken together, these results suggest that hepatic colonization is brought about by organ-specific tumour cell migration and chemotactic stimuli within the liver. Downstream signalling of both integrins and chemokine receptors involves small Rho-GTPases. Corresponding to the increased cell motility of cancer cells, it was found that RhoA, Rac1 and Cdc42 are dramatically overexpressed in breast cancer, but also in colon and lung cancer compared to corresponding normal tissues [25]. These GTPases are involved in the detection of chemotactic gradients and the formation of cell polarity, and they also represent importartant regulators of actin, modulating the assemby and disassemby of actin filaments. Their activation leads to stress fibre formation, lamellipodial protrusions, membrane ruffling and directed cell movement [26].

We postulated that tumour cell extravasation into host organs is a result of the specific availability of chemokine responses within the target organ's microenvironment and the organ's specific composition of ECM components. We observed tumour cell arrest within the liver intravitally and investigated the adhesive and migratory properties of breast cancer cells in response to various ECM components in vitro, thus analysing the role of the CXCL12/CXCR4 axis in the initial steps of the metastatic cascade of host organ colonization.

## Materials and Methods

### Reagents

Phycoerythrin (PE)-conjugated anti-human CXCR4 antibodies were obtained from R&D Systems (Wiesbaden, Germany). Unlabelled, function-blocking anti-human CXCR4 antibody was a kind gift from A. Müller, Düsseldorf, Germany. Anti-human RhoA antibody was purchased from Santa Cruz (Santa Cruz, CA, USA), anti-human Rac1 from BD Pharmingen (San Jose, CA, USA) and anti-human Cdc42 antibody was obtained from Cell Signaling (Danvers, MA, USA). For integrin subunits the following antibodies were used: β1, β4, α2, α3 (all from Chemicon, Hofheim, Germany), α1 (upstate biotechnology, Hamburg, Germany), α5 (Serotec, Eching, Germany) and α6 (gift from J. Eble, Münster, Germany). Alexa Fluor 488 and 546 labelled secondary antibodies, phalloidin, CalceinAM and Hoechst 33342 were purchased from Molecular Probes/Invitrogen (Karlsruhe, Germany). Human recombinant chemokine SDF1α/CXCL12 was obtained from R&D systems (Wiesbaden, Germany).

Glutathione-Agarose Rac/Cdc41 PAK-1 PBD beads and Rho Rhotekin RBD beads were obtained from Upstate/Millipore (Eching, Germany). ECM components C I, FN and LN were purchased from Sigma-Aldrich (Saint Louis, Missouri, USA). All other chemicals were purchased from Sigma or Roth (Karlsruhe, Germany).

### Cell lines and culture conditions

Subclones of the MDA-MB-231 cells (originally obtained from the ATCC) were provided by M. Kochetkowa, Adelaide, Australia. These cells were originally derived from a 51 years old Caucasian female and are invasive and tumorigenic in nude mice. MDA-MB-468 cell line was purchased from ATCC (Manassas, VA) and was originally derived from a pleural effusion of a 51-year old black female patient with metastatic adenocarcinoma of the breast. MDA-MB-231 cell line was maintained in RPMI 1640 or DMEM medium (Gibco/Invitrogen, Karlsruhe, Germany) containing 10% foetal bovine serum (FBS, Gibco) without antibiotics. Two clones with shRNA-mediated CXCR4 reduction were also provided by M. Kochetkowa. These clones (MDA-MB-231-19 and MDA-MB-231-27) were obtained using CXCR4 shRNA-expressing constructs 5-gatctGGTGGTCTATGTTGGCGTCTGttcaagaGACAGACG CCAACATAGACCACCtttttta-3 and 5-agcttaaaaaaGGTGGTCTATGTTGGCGTCTG tctcttgaacagacgccaacatagaccacca-3 (21-nucleotide CXCR4 at position 470–490 of human CXCR4 cDNA). [21], [27] MDA-MB-468 cells were cultured in DMEM medium (Lonza, Verviers, Belgium) containing 10% FBS and L-glutamine (Gibco/Invitrogen, Karlsruhe, Germany) without antibiotics. The cells were starved overnight in serum-free media before their application in experiments. After trypsinization, the cells were resuspended in serum-free adhesion medium (containing 1% bovine serum albumin) for reconstitution of surface proteins prior to experimentation.

### Transwell assay

The breast cancer cells were added to FN, LN or C I-coated transwell inserts with 8 µm-pore-size (Nunc/Thermo Fisher Scientific, Rockford, IL, USA). The cells were suspended into the upper chamber at a final concentration of 0,7×10^5^ cells/ml in 500 µl adhesion medium. After 60 min adhesion time, CXCL12 in concentrations 25, 50 or 100 ng/ml were added to the lower chamber. Unstimulated cells served as negative control. For diffuse stimulation CXCL12 at equal concentrations was added in the lower and upper chamber.

After 4 or 16 h of incubation, the cells on the upper surface of the filter were removed by wiping with Q-tips, and the migrated cells on the lower side were fixed with formalin and stained with crystal violet and haematoxylin. Cellular transmigration was enumerated in 16 standardized microscopic fields per membrane.

To test the specific chemotactic response of the cells to CXCL12 the CXCR4 receptor was blocked using a neutralizing mouse-anti-human CXCR4 antibody (kind gift of A. Müller, Düsseldorf, Germany). The antibody was added to the upper chamber at concentrations of 5 and 10 µg/ml and migration of the cells was observed for 4 or 16 h.

### Static adhesion assays

Microtiter plates (96 wells, Greiner BioOne, Frickenhausen, Germany) were coated with C I (10 µg/ml), FN (10 µg/ml), LN (10 µg/ml) or 1% BSA (negative control). Blocking of nonspecific binding sites was performed with 1% BSA for 30 min. After reconstitution of cell surface proteins in adhesion medium for 45 min, cells were resuspended in adhesion medium at a final concentration of 1×10^6^ cells/ml and seeded to the coated wells. The cells were stimulated with CXCL12 with concentrations of 25, 50 and 100 ng/ml. After 30 or 60 min adhesion time cells were washed, fixed with formalin and then stained with crystal violet for 15 min. The absorbance was measured at 630nm using a spectrophotometer. All experiments were performed in triplicates and repeated at least three times.

### Time lapse microscopy

The cells were added to ECM-coated culture dishes for 20 min. After washing adhesion medium containing CXCL12 at different concentrations was added. The cells were observed for 60 min under a time lapse video microscope (Nikon, Düsseldorf, Germany). For quantification of cell motility cell tracking was done using software packages Cell∧D.

### Intravital microscopy

Intravital microscopy was performed as previously described [6], [7]. The adequacy and reliability of this model in the investigation of early interactions between circulating tumour cells and the hepatic microcirculation was confirmed previously [28]. Briefly, Sprague Dawley rats (200 to 250 g) (Charles River, Sulzfeld, Germany) were cared for in accordance with standards of the German Council on Animal Care, under an approved protocol of the local animal welfare committee (Landesamt für Naturschutz, Umweltschutz und Veterinärmedizin: LANUV G84/2002). Rats were anesthetized using inhalation of isofluorane (Curamed, Karlsruhe, Germany). Permanent catheters were introduced into the left heart via the carotid artery and the right heart via the jugular vein. After a wide median laparotomy was performed, the left liver lobe was careful mobilized without disturbing hepatic microcirculation. Using a heated operating table, animals were fixed under an upright microscope and positioned on their left side. This positioning allowed a partial luxation of the mobilized left liver lobe that was placed on a specific holder to investigate its lower surface. During the experiments the liver was continuously irrigated with isotonic saline solution.

An upright epifluorescence microscope (Zeiss, Oberkochen, Germany) was used with a 20-fold objective that was located over a glass slip covering the organ surfaces. The microscope was connected with a video enhancer-zoom lens system and a low-light charge-coupled device video camera (Peiper, Düsseldorf, Germany) allowing real-time imaging via a separate monitor. Fluorescence images were recorded using timer-containing S-VHS video system for further analysis.

### In Vivo Observation of Metastatic Tumour Cell Adhesion and Extravasation

For intravital observation of adhesive interactions between circulating tumour cells and the host organ microcirculation, single cell suspensions of CalceinAM fluorescence-labelled tumour cells (1×10^6^) were injected intra-arterially within 60 sec. Previously [6], [7], we have shown that the route of cell application (left heart, right heart, portal vein) did not influence the adhesive or migratory behaviour within the liver sinusoids. This technique did not interfere with cardio-circulatory or pulmonary functions of the animals.

Off-line analysis was used to determine tumour cell behaviour within the target organs as previously described [24], [29]. Various parameters were used for further investigation and semiquantitative analysis of these interactions. A semiquantitative analysis of tumour cell adhesion and extravasation was performed throughout a 30 min observation period, and the numbers of adherent cells were counted for each of the 5 min intervals. Using a standardized procedure, all fields were analysed in each observation period and average numbers of adherent cells, migrated cells, and total cells observed were counted. The numbers provided represent the total numbers of cells within 30 microscopic fields for each 5 min period. Numbers of arrested cells represent the total of adherent and extravasated cells. Relative migration rates were calculated as percentages of cells within the host organ parenchyma in relation to the numbers of arrested cells.

### GTPase activation assay

Non-adherent cells (10^7^) in suspension unstimulated or stimulated with CXCL12 (25, 50, 100 ng/ml) for 15 min were washed once with ice cold PBS and lysed with 50mM Tris-HCl pH 7.4, 150 mM NaCl, 1% NP40, 0.5% deoxycholate, 0.1% SDS, 5mM EDTA and 1 µl inhibitor cocktail (Sigma) per 1 ml lysis puffer. Alternatively, adherent cells were seeded at different ECM proteins (C I, FN and LN) for 60 min to exclude interference with initial adhesive behaviour and subsequently stimulated with CXCL12 in a similar manner. As controls poly-l-lysine (PLL for non-integrin mediated adhesion) and BSA (negative control) were used.

Lysates were clarified by centrifugation at 14,000xg for 5 min and stored at −80°C. After protein quantitation and standardization 8 µl Rho Assay Reagent (Rhotekin RBD glutathione agarose beads) or 10 µl Rac/Cdc42 assay reagent (PAK-1 PBD agarose conjugate) were added to 1000 µg total protein and incubated for 45 min at 4°C. Samples were washed three times with magnesium-containing lysis puffer (25 mM HEPES pH 7.5, 150 mM NaCl, 1% Igepal CA-630, 10% Glycerol, 10mM MgCl_2_, 1mM EDTA) and 1 µl inhibitor cocktail per 1 ml lysis puffer. Agarose beads were resuspended in 4x Laemmli sample puffer and boiled for 5min. Total Rho, Rac-1 or CDC42, respectively, served as loading control in each of the experiments. The lysates were loaded on 12% polyacrylamide gels, then transferred to PVDF membranes and GTPases finally detected with rabbit anti-RhoA antibody (Santa Cruz), mouse anti-Rac antibody (BD Biosciences Pharmingen) or rabbit anti-Cdc42 antibody (Cell Signalling). Bands were visualized with enhanced chemiluminescence (Millipore, Schwalbach, Germany). Quantitative densitometry analysis was performed using ImageJ densitometry software (version 1.6, NIH, Bethesda, MD) and selected bands were semi-quantified based on their optical densities.

### Immunofluorescence staining

The cells were added to C I, FN, LN or Poly(L)lysine (PLL)-coated chamber slides, incubated for 60 min and then stimulated using different chemokine concentrations (25, 50 and 100 ng/ml) for different time intervals (5, 15 and 30 min). Subsequently, cells were washed and fixed using 4% paraformaldehyde. For intracellular staining, the cells were blocked and permeabilized using PBS containing 1% BSA and 0, 1% Triton X100, whereas permeabilization was not performed to achieve cell surface staining. Cells were incubated for 30 min with anti-human CXCR4 antibodies and subsequently with Alexa Fluor conjugated secondary antibody for additionally 30 min. For actin filament staining phalloidin Alexa Fluor 488 and for nuclear staining Hoechst 33342 was used.

Imaging was performed as combination of 3D-fluorescence reconstruction and phase contrast microscopy using a Nikon Eclipse TE2000 microscope. For each substrate 20 representative CXCR4 clusters were evaluated regarding their localization at the cell, their size and shape. The size of CXCR4 clusters was calculated using software packages Cell∧D (Olympus, Münster, Germany).

### Flow Cytometry

Cells were fixed with 4% paraformaldehyde and then washed and resuspended in PBS containing 0.5% BSA. After this the cells were incubated for 45 min with PE-conjugated anti chemokine antibodies (R&D systems). After washing the integrin or chemokine receptor surface expression was measured using a FC500 flow cytometer (Beckman Coulter, Krefeld, Germany). After gating of the cell population, the mean fluorescence intensities (MFI) of the antibody-stained cells were detected and the relative amounts of positive cells were calculated using the flow cytometer software.

### Statistical analysis

Statistical analysis was performed using the SPSS V.14 (SPSS Inc., Chicago, IL) statistical program. Data were shown as mean±SD. For comparison of different parameters between the treatment groups p-values were calculated according to the Scheffé-test (ANOVA post-hoc-test) for dependent or independent samples as appropriate. For other analyses Student's t-test has been used. Significant differences were accepted for p<0.05.

## Results

### Chemokine-induced cell migration in vitro

**Figure 1:**
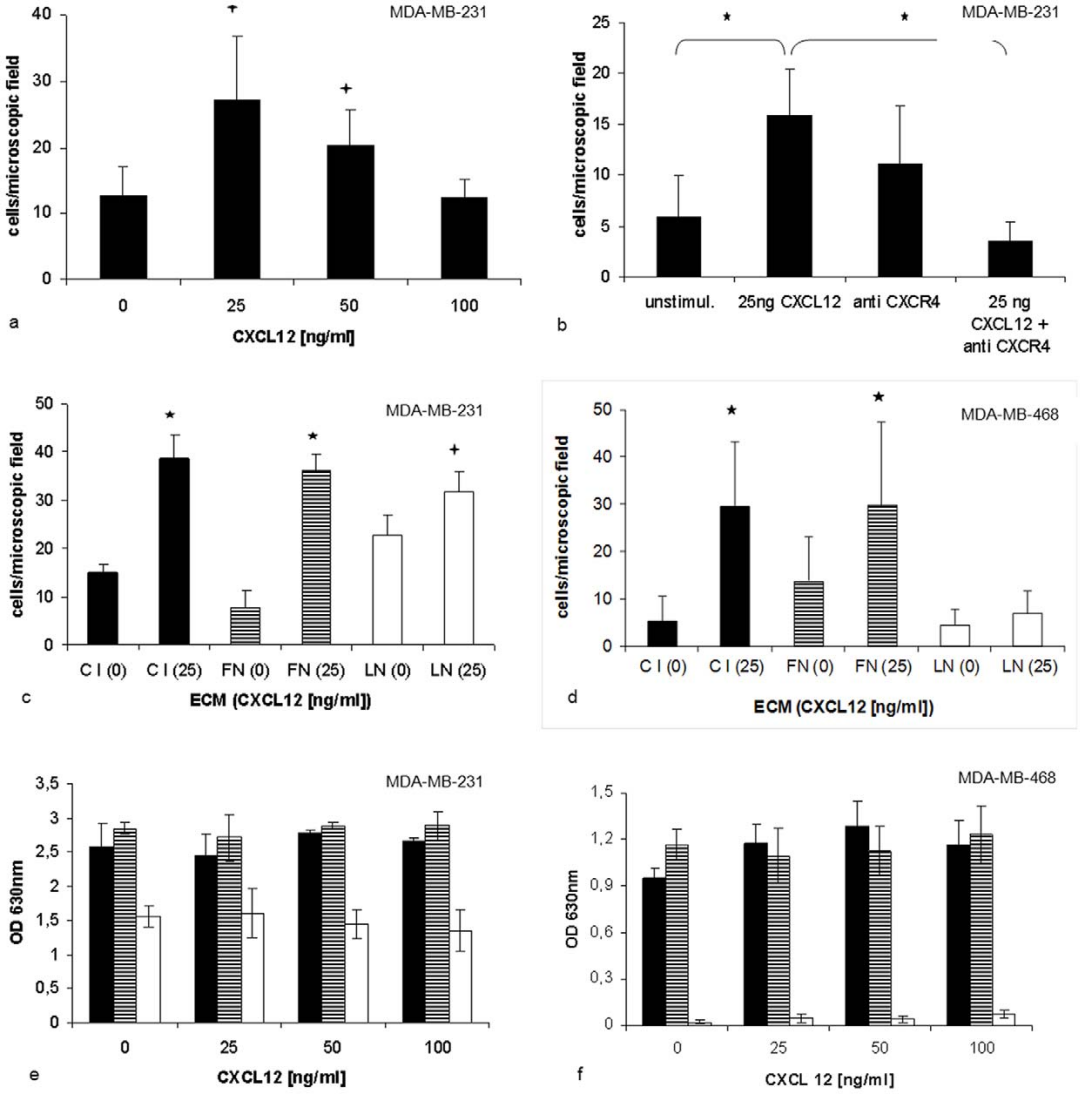
CXCL12 stimulated cell adhesion and migration. Transwell migration assays were performed for 16 h using different gradients of CXCL12 (0–100 ng/ml). (a) Bell-shaped response is shown for MDA-MB-231 cells at C I. (b) This response was specific for CXCL12 and could be reversed by inhibitory anti-CXCR4 antibodies in these cells. A slight agonistic effect of the anti-CXCR4-mAb was observed. (c) In MDA-MB-231 cells this response (25 ng/ml CXCL12) occurred at all ECM components, but to different extent. (d) In contrast, in MDA-MB-468 cells stimulated migration (25 ng/ml CXCL12) was found at C I and FN, but not at LN. The values are reported as means ± SD of three independent experiments (* p<0.001 and +p<0.05). Cell adhesion: (e) MDA-MB-231 and (f) MDA-MB-468 cells were plated at C I (▪), FN (≡), LN (□). Binding to BSA coated surfaces was used as background control. Cells were treated with 25, 50 or 100 ng/ml CXCL12 or left untreated (0 ng/ml). After 30 min adhesion time absorbance was measured as optical density (OD) at 630 nm. CXCL12 did not significantly modify cell adhesion of both cell lines, but adhesion properties were depended from the adhesive substrates with high adhesion rates at C I and FN compared to low or absent adhesion at LN.

To examine chemotactic migratory responses to chemotactic stimuli MDA-MB-231 and MDA-MB -468 cells were exposed to different CXCL12 gradients (0, 25, 50 and 100 ng/ml) in Transwell chambers coated with different ECM components. Spontaneous migration without chemotactic gradients was found in both cell lines at basal levels. Bell-shaped concentration dependence showed maximum effects at 25 ng/ml at C I and FN (p<0.05). Higher concentrations (100 ng/ml) resulted in migration rates at these ECM components that were comparable to unstimulated cells. (fig. 1a). In order to confirm specific responses, cells were additionally treated with an inhibitory anti-CXCR4 antibody. In unstimulated cells this resulted in slightly increased migration rates suggesting partial agonistic activity of the antibody. In contrast, in stimulated cells, anti-CXCR4 completely blocked migration and only basal levels of migration were detected. (p<0.001; fig. 1b) Finally, migration rates were compared in the presence of different ECM components. Chemotactic response to CXCL12 significantly increased 3–4 fold at C I and FN in both cell lines (p<0.001). In contrast, at LN, a slight but significant (p<0.05) increase was induced in MDA-MB-231, but not MDA-MB-468 cells. (fig. 1cd+)

### In Vitro Tumour Cell Adhesion

We analysed whether cell adhesion properties to the proposed interacting ECM components could be modified by CXCL12 in vitro. During adhesion to C I, FN or LN for 30 or 60 min, tumour cells were treated with CXCL12 at concentrations of 25, 50 and 100 ng/ml or remained untreated. Under untreated conditions, both cell lines showed high adhesion rates at C I and FN, but only moderate (MDA-MB-231, fig. 1e) or weak cell adhesion (MDA-MB-468, fig. 1f) when LN was present. These differences were expected due to the differing expression of LN-receptors at the cells' surfaces (Table 1). The presence of CXCL12 did not significantly modulate this static cell adhesion at any of the ECM components in both cell lines. (fig. 1e+f) In addition, stimulation with 25ng/ml CXCL12 for 15 min in nonadherent single cell suspensions of both cell lines did not affect the integrin surface expressions. (data not shown)

**Table 1:** Cell surface expression of chemokines receptors.

	MDA-MB 231 wt	MDA-MB 231-19	MDA-MB 231-27	MDA-MB 468
neg	0.9	0.7	0.5	0.3
IgG control	3.5	4.2	2.7	14.9
CCR 3	72.6	76.1	75.2	94.2
CCR 7	18.3	26.7	37.4	70.6
CXCR 1	82.7	79.8	81.9	94.2
CXCR 2	5.2	6.6	7.5	56.7
CCR 1	1.6	1.8	1.0	2.0
CCR 2	1.9	1.9	1.3	2.1
CCR 6	33.9	35.1	33.7	89.4
**CXCR 4**	**84.5**	**39.8**	**41.5**	**78.7**
CXCR 5	78.7	75.1	77.9	91.3
CCR 5	40.1	58.6	63.1	88.2
CXCR 3	85.4	86.4	80.2	93.2

Cell surface expression was analysed by flow cytometry. shRNA transfection resulted in a reduction of CXCR4 expression in MDA-MB231-27 cells. Negative and IgG controls are given as examples for the controls that have been used in each measurement. IgG controls were subtype-specific.

### Expression of CXCR4 chemokine receptors

**Figure 2:**
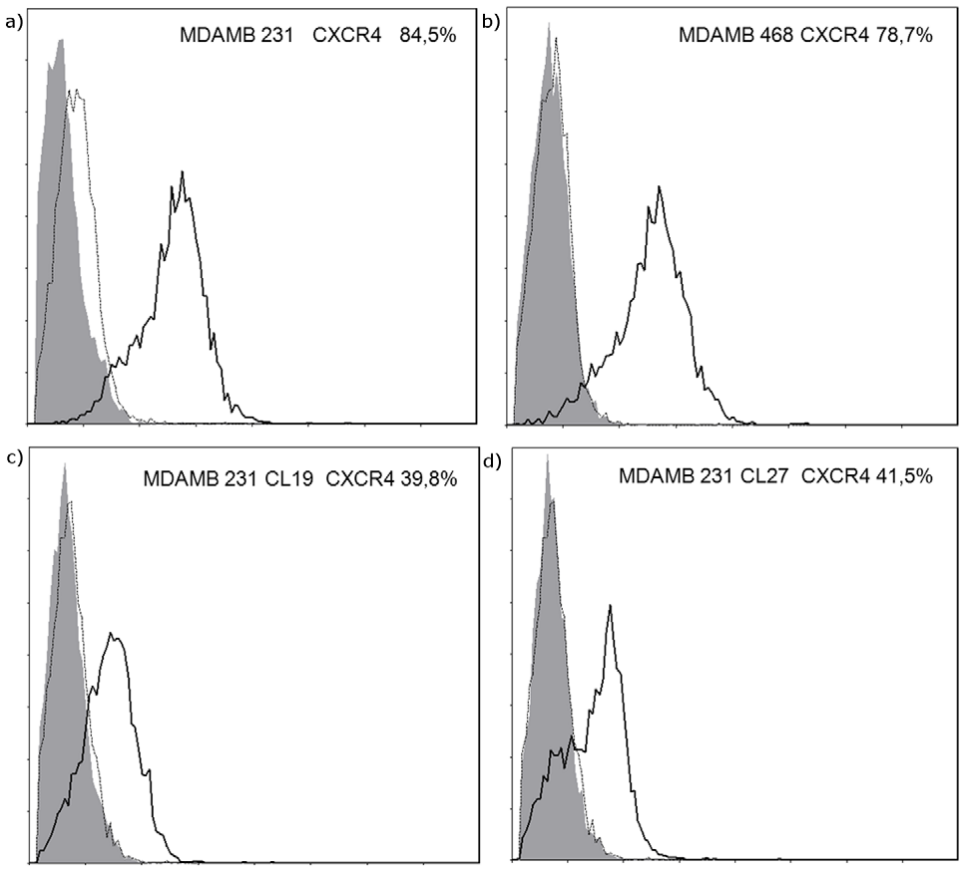
Flow cytometry analysis of breast cancer cells. The cell surface expression of CXCR4 (⁃) was found in different breast cancer cell lines MDA-MB-231 (a) and MDA-MB-468 (b). Downregulation of CXCR4 expression in clones MDA-MB-231 Cl 19 (c) and MDA-MB-231 Cl 27 (d). (isotype control IgG2b: ••••; negative control:▪).

The highly metastatic MDA-MB-231 cells expressed CXCR4 in 84.5%, whereas the less metastatic MDA-MB-468 cells showed 78.7% expression. The two clones MDA-MB-231-19 and MDA-MB-231-27 had ∼40% residual CXCR4 expression as estimated by flow cytometry (Table 1, fig. 2).

### CXCL12 affects the motility of breast cancer cells

Our previous observations [24], [30] of chemotactic responses and ECM-dependence of colon carcinoma cell extravasation into the liver suggested interactions between ECM composition and chemotaxis. The results of this study confirmed these observations. Thus, we studied the dependence of short term chemokine stimulation and cell motility using single cell fluorescence-assisted time lapse microscopy. The cells were observed for 1 h in 30 sec intervals.

**Figure 3:**
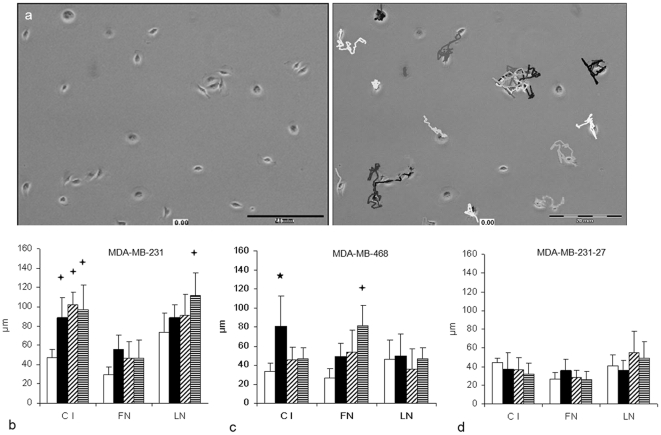
CXCL12 increases cell motility dependent from the ECM. (a) Cells that were attached to different ECM components were observed for one hour using time-lapse video microscopy. The length of their moving path was determined using a cell tracking software (Cell∧D®). (b) MDA-MB-231 and (c) MDA-MB-468 cells showed different patterns of chemokine stimulated motility (□ unstimulated; ▪ 25ng/ml; ///50ng/ml; ≡ 100ng/ml CXCL12). Highly metastatic MDA-MB-231 cells were more motile at CI and LN (*p<0.001) with a concentration-dependent stimulation (+p<0.05) of their motility that was not observed at FN. In contrast, low-metastatic MDA-MB-468 cells were less motile at C I and LN, but showed a concentration-dependent stimulation at FN. (d) In CXCR4-knock-down cells (MDA-MB-231-27) these CXCL12-dependent stimulating effects were not found. The results are shown as mean ± SD for three independent experiments. For statistical analysis ECM dependent migration was compared for all three ECM components and concentration dependence of CXCL12 was compared to unstimulated cells. Significances for ECM dependent cell motility are not marked for improved readability.

For quantification of these effects we initially determined cell surface areas, their circumferences, various cell diameters, length of moving path and the numbers of membrane ruffles over the time with or without CXCL12 stimulation. All of these parameters showed high variability between the cells (data not shown), but length of the motility path was the single parameter with reproducible effects that was used for further quantitative analysis. Using a cell tracking software, time-dependent path lengths of the cells were quantified (figure 3a).

If cells remained without chemokine stimulation, both cell lines showed comparable patterns of basic cell motility, but MDA-MB-231 cells were more motile than MDA-MB-468 cells with longer lengths of path during the observation period. In the presence of CXCL12, cells plated at C I had a similar increase in motility with a slightly different concentration-dependence in both cell lines (fig. 3b+c). Comparable to transwell migration, only MDA-MB-231 cells were responsive to CXCL12 at LN, whereas at FN, only MDA-MB-468 cells showed stimulation of motility. The CXCL12-dependent stimulating effects were not found in CXCR4-knock-down cells (MDA-MB-231-27). (fig. 3d)

MDA-MB-231 cells plated at C I developed distinct lamellipodia. If stimulated with CXCL12, the cells were more motile and showed directed movement. In the presence of FN, these cells formed smaller lamellipodia but formed protrusions especially using 25ng/ml CXCL12. When MDA-MB-231 cells migrated at LN, numerous filopodia and spindle-like cell forms were observed (see Video S1).

Low metastatic MDA-MB-468 cells developed small filopodia (microspikes) if seeded at C I or FN. Lamellipodia were not formed at C I, but cells appeared stretched when FN was present. In the presence of high concentrations of CXCL12 (100 ng/ml), the cells partially started lamellipodia formation. In contrast to MDA-MB-231 cells, MDA-MB-468 cells did not spread at LN and formed only short filopodia (see Video S2).

### CXCL12 induces tumour cell migration into the liver parenchyma

**Figure 4:**
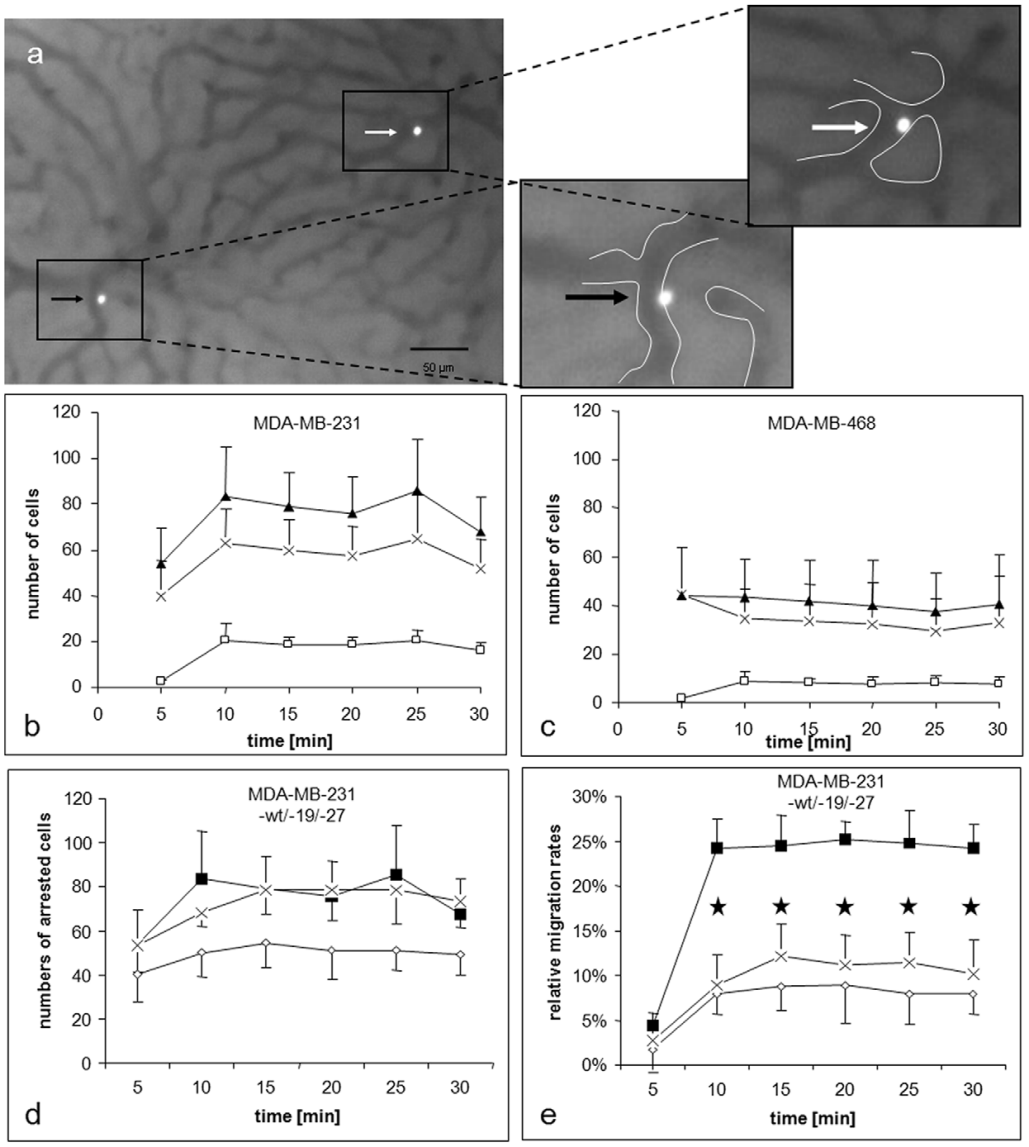
In vivo migration of breast cancer cells within the liver sinusoids. Single cell suspensions of fluorescence-labelled tumour cells were injected into Spague-Dawley rats. (a) Example of an adherent cells (white arrow) and a cell starting to migrate (black arrow) into the liver parenchyma. Magnifications show location of the cells in relation to marked sinusoid-parenchyma borders. Intravital microscopy was done to analyse adhesion and migration properties of MDA-MB-231 (b) and MDA-MB-468 cells (c). Thirty microscopic fields were analysed in 5min observation periods for semiquantitative analysis of adherent (x) and migrated cells (□). The total numbers of arrested cells (▴) were calculated. **CXCR4 inhibition decreased tumour cell migration in vivo.** The MDA-MB-231 cells were transduced with shRNA to inhibit CXCR4 expression. Two clones 19 (◊) and 27 (x) were tested for in vivo adhesion (d) and migration (e) in the rat liver and compared with untreated cells (▪). Transfected cells showed significantly (*p<0.001) decreased relative migration rates into the liver parenchyma but cell adhesion within the hepatic microcirculation was only slightly influenced by CXCR4 reduction. Relative migration rates were based on the number of arrested cells and were calculated as described [26].

The in vitro results suggested that chemotactic CXCL12/CXCR4-signaling is a crucial determinant of site-specific tumour cell extravasation into the liver that was further analysed using the in vivo model for hepatic tumour cell colonization. Breast cancer cells showed specific adhesive and migratory properties as previously described for other tumour entities [6], [7], [23], [26]. Differentiation between adherent and extravasated cells enabled semiquantitative analysis. All arrested cells did not completely occlude the vessel lumen, suggesting specific adhesive interactions with the sinusoidal vessel wall. (fig. 4a) Using highly metastatic MDA-MB-231 cells, significantly (p<0.05) higher numbers of cells arrested within the hepatic sinusoids compared to less metastatic MDA-MB-468 cells (MDA-MB-231: 67–85 cells/interval; MDA-MB-468: 37–43 cells/interval). In addition, MDA-MB-231 cells extravasated at similar relative migration rates into the liver parenchyma. (MDA-MB-231: 19–25%; MDA-MB-468: 20–23%). (fig. 4b+c)

Since CXCL12 is specifically expressed within the sinusoidal structures [28] we investigated whether this chemokine and its receptor CXCR4 are involved in this behaviour. To validate the CXCL12-induced breast cancer cell migration in vivo MDA-MB-231 cells with shRNA-mediated reduction of CXCR4 (MDA-MB-231-19 and MDA-MB-231-27) were compared with wild-type cells. In both clones this inhibition of CXCR4 receptor expression did not affect adhesion of tumour cells within liver sinusoids (fig. 4d) but significantly (*p<0.001) decreased relative migration rates (fig. 4e).

This observation raised the question whether the rapid extravasation is mediated by chemotactic responsiveness of breast cancer cells within the hepatic micro­environment or by different integrin expression at their surfaces.

### Expression of integrins

In order to exclude differences in the cell surface expression of both cell lines, flow cytometric analysis of CXCR4 and integrin expression was performed. The cell surface expression of most integrin subunits relevant to the ECM components used in this study were present in similar amounts in both cell lines. The major difference between the cell lines was observed for ligands of LN (α3, α6 and β4) that were detected in both MDA-MD-231 and MDA-MB-468 cells, but were less present in the latter. Integrin receptors for C I or FN binding were available in both cell lines. (Table 2) Since both cell lines showed similarly high CXCR4 expression levels, functional responsiveness of this receptor was tested.

**Table 2:** Expression of integrin subunits at cell surfaces.

Integrin subunits	MDA-MB-231	MDA-MB-468	Ligands
Neg.	0.5	0.3	
IgG control	1.6	0.9	
α1	93.7	96.0	C I, LN
α2	24.9	98.6	C I, LN
α3	99.8	96.5	LN 5+10
α5	87.9	53.7	FN
α6	81,4	33,8	LN 1+5
αv	97.2	99.7	FN
β1	98,6	99,2	C I, FN, LN
β4	68,7	48.5	LN

Cell surface expression was analysed by flow cytometry. The major difference between the cells was observed for integrin ligands of LN. Negative and IgG controls are given as examples for the controls that have been used in each measurement. IgG controls were subtype-specific.

### Signalling responsiveness of CXCR4

We investigated whether binding of the ligand CXCL12 could activate CXCR4 signalling and whether these responses were be modulated by ECM binding. CXCR4 responsiveness was tested using GTPases RhoA, Rac1 and Cdc42 as markers for its downstream signalling. As mentioned above, FN and C I, but not C IV or LN were found to mediate cell adhesion and migration within the liver [24]. Therefore, we specifically focussed on C I and FN, respectively. Since LN is not involved in these processes and was found only in small quantities within the liver, we used this ECM component as control.

**Figure 5:**
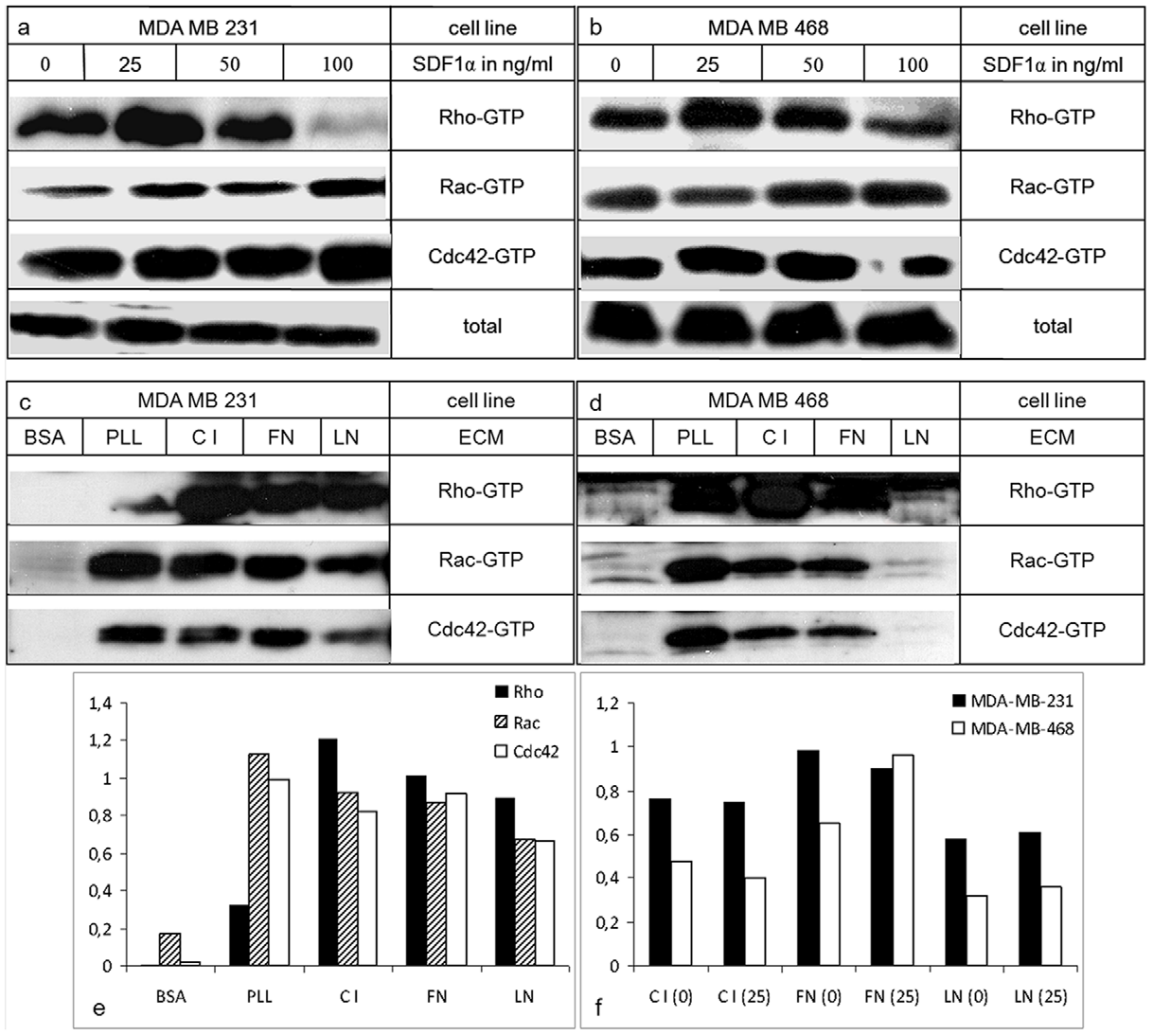
CXCL12 and ECM-induced activation of small Rho GTPases. Cells were treated with different concentrations of CXCL12 in single cell suspensions (a+b) or plated at various adhesive substrates (c+d). Level of activation of RhoA, Rac1 and Cdc42 assessed by pull down assays of the GTP-bound forms were determined in MDA-MB-231 (a+c) and MDA-MB-468 cells (b+d). As example for total GTPase detection independent from phosphorylation status in each experiment Total-Rho is shown for (a) and (b). These loading controls were used for standardization of optical densities (e+f). RhoA and Rac1 activation increased in a dose-dependent manner, but Cdc42 remained at baseline levels. If MDA MB 231 (c) and MDA MB 468 (d) cells were plated at C I, FN, LN, PLL and BSA differences in ECM dependent activation of GTPases were observed. Comparing relative optical densities these matrix dependent differences were confirmed (e). Synergistic activation between chemokine stimulation and ECM binding, such as demonstrated for Rho (f), were not found.

Initially, CXCL12 treatment was performed in single cell suspensions to show integrin and adhesion independent activation of the GTPases. Basal levels of Rho-GTP, Rac-GTP and Cdc42-GTP in untreated cells were detected in both cell lines. Treatment with different concentrations of CXCL12 resulted in increased activation of RhoA in MDA-MB-231 (+40%) and MDA-MB-468 (+60%) cells compared to untreated cells. (fig. 5a+b) Both cell lines showed bell-shaped concentration dependence with a maximum at 25 ng/ml. Rac1 activation was increased up to two-fold at higher CXCL12 concentrations of 50 or 100 ng/ml in both cell lines, whereas relevant CXCL12-induced Cdc42 activation was not observed. (fig. 5a+b) This activation reached a maximum at 15 min after stimulation and subsequently dropped to basal levels after 30–45 min. (data not shown)

For analysis of integrin-dependent activation of the Rho GTPases, tumour cells were plated at C I, FN or LN. MDA-MB-231 cells (fig. 5c) showed Rho, Rac and Cdc42 activation if cells were plated at C I and FN, but lesser activation at LN. Using MDA-MB-468 cells, (fig. 5d) the GTPases showed less activation in the presence of C I and FN compared to MDA-MB-231 cells. The most important difference however, was the almost complete lack of GTPase activation after seeding MDA-MB-468 cells at LN. As expected, BSA induced no activation of GTPases in both cell lines. PLL served as an integrin-independent control. Optical densities were standardized against the Total-GTPase counterparts and these relations were confirmed in all experiments as shown for one example in fig. 5e.

In order to assess a potential synergism between integrin-mediated and chemokine-induced activation of the GTPases, ECM-seeded cells were stimulated with different concentrations of CXCL12. This resulted in comparable GTPase activation between unstimulated and stimulated cells. Only FN induced a slight synergistic increase of Rho in MDA-MB-468, but not MDA-MB-231cells (fig. 5f).

### ECM induced reorganization of CXCR4 cell surface distribution

**Figure 6:**
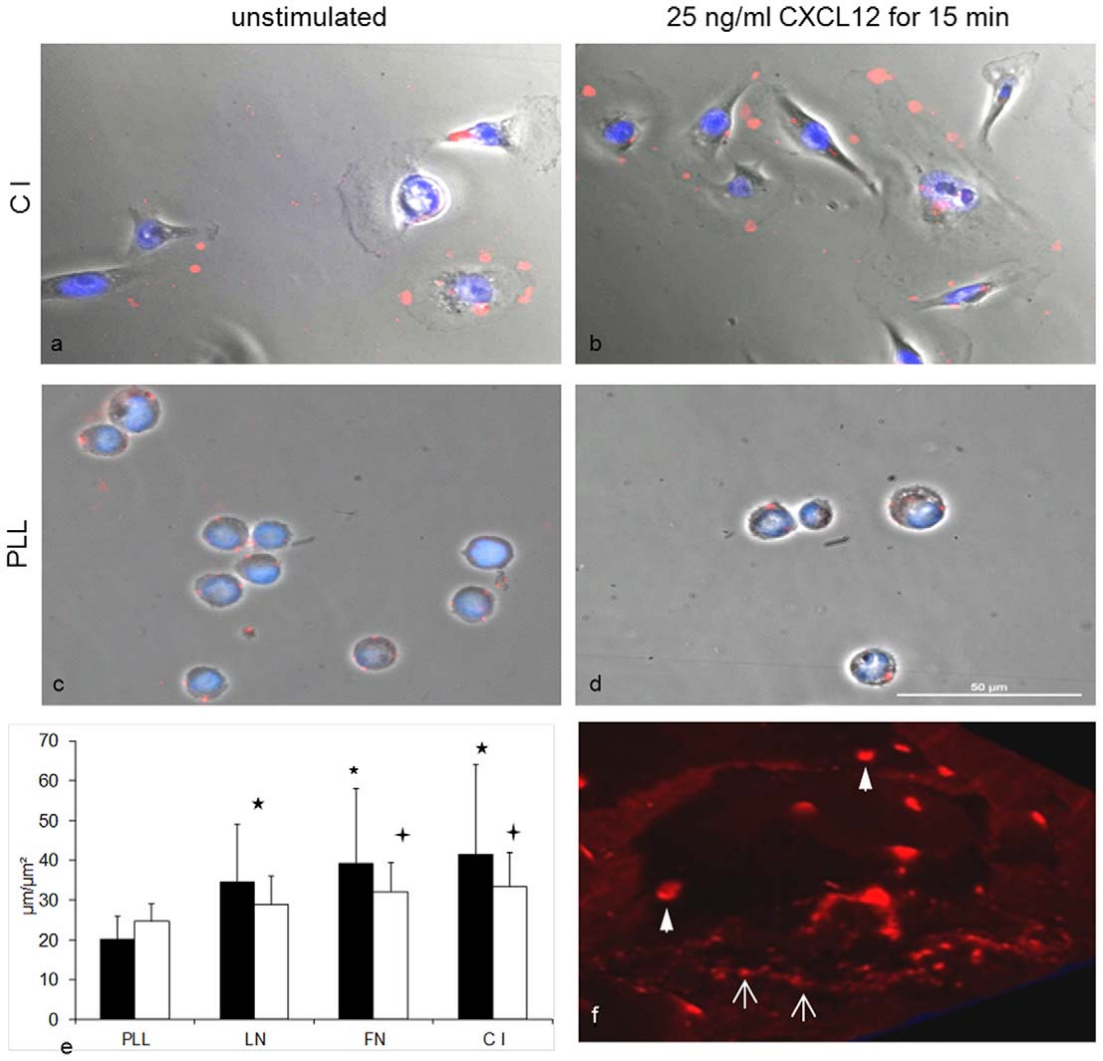
Clustering of CXCR4 at tumour cell surfaces. CXCR4 was visualized in wild-type MDA-MB-231 cells using fluorescence-labelled antibodies (red). Nuclei were counterstained (blue). Scale 50 µm. Figures were taken 15 min after CXCL12 stimulation and merged with phase-contrast for cellular location of the CXCR4. In both cell lines similar distributions of CXCR4 were observed. MDA-MB-231 cells at C I (a+b; as integrin dependent) and PLL (c+d; as integrin independent control) are shown as examples. Cell spreading at ECM components induced formation of large CXCR4 clusters at lamellipodia and pseudopodia (a), whereas in cells adherent at PLL only small dot-like CXCR4 clusters were observed (c). Presence of 25 ng/ml CXCL12 (C I: b, PLL: d) did not result in alterations of the size or localization of these clusters. (e) The sizes of 20 clusters in at least 10 cells per experiment were evaluated for each adhesive substrate. Areas (µm^2^) of these clusters (▪) and their circumference (µm, □) were significantly higher in cells that were spread in an integrin-dependent manner at C I, FN or LN compared to unspread cells at PLL (* p<0.001; +p<0.05). (f) Larger clusters (▸) and dot-like CXCR4 structures (→) were found with a preference of the dot-like structures in the direct neighbourhood of leading adhesive boundaries.

Lateral redistribution of cell surface receptors, such as integrins and growth factor receptors, is known as a mechanism for their activation and can create synergistic functional effects. Since an interaction between different ECM components and CXCR4 was postulated, we further investigated the distribution of this receptor on the surface of adherent cells using immunohistochemical staining. After seeding at ECM components, CXCR4 was detected in clusters in both cell lines. Initially, small clusters were found in cells in the presence of all ECM components including PLL without preference of certain localizations. (not shown) During cell spreading on ECM components, CXCR4 clusters were relocated to lamellipodia (predominantly seen in FN) or pseudopodia (predominantly seen in C I) within 5–30 min. In contrast, in cells adherent to PLL, only small CXCR4 clusters were observed without changes over time. (fig. 6a–d) The size of the clusters significantly increased in cells spread at ECM components compared to PLL-mediated cell attachment (* p<0.001; +p<0.05; fig. 6e). Corresponding to the lower level of integrin ligand expression for cell adhesion at LN, spreading at this adhesive substrate and formation of CXCR4 clusters was less pronounced than for FN and C I.

**Figure 7:**
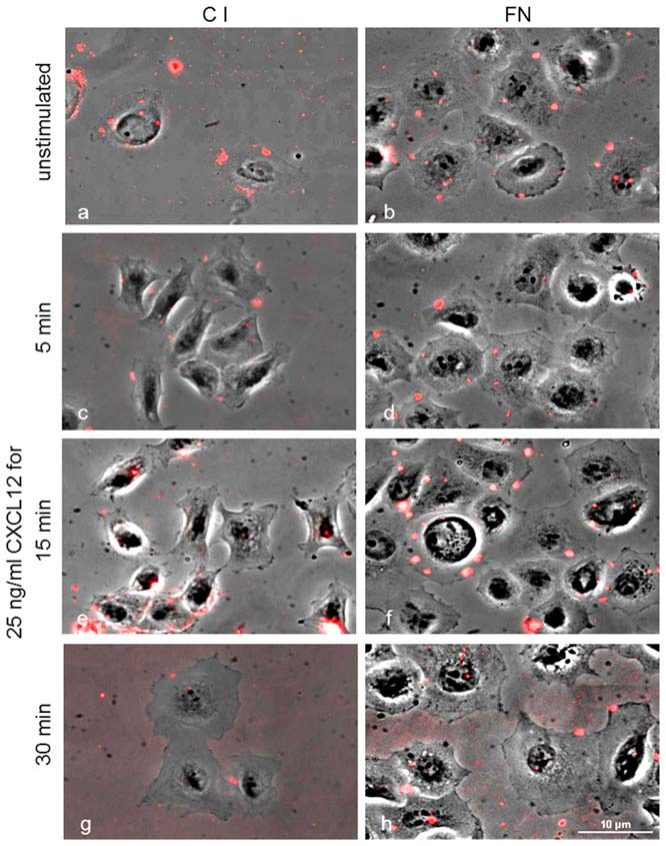
Kinetics of CXCR4 cell surface expression. MDA-MB-231 cells were seeded at C I (left column) or FN (right column) and stimulated with CXCL12 for up to 30 min. Fixed cells without stimulation (a+b) or with CXCL12 stimulation (25 ng/ml) for 5 min (c+d), 15 min. (e+f) or 30 min (g+h) were stained for surface expression of CXCR4. The number of large CXCR4 clusters was reduced after 30 at cells in a comparable manner for both ECM components. Scale 10 µm

Using 3D-reconstruction, we found that larger clusters were located above the leading edges slightly apart from the adherent cell regions, whereas small dot-like clusters were located directly at the leading edges (fig. 5f). The presence of CXCL12 did not result in differing sizes or localizations of these clusters compared to unstimulated cells in both cell lines. In contrast, using PLL for non-integrin-mediated cell attachment, CXCR4 was detected without signs of special distribution (fig. 6d). Co-localization of CXCR4 clusters with focal adhesion kinase was used to stain redistribution towards integrin-mediated focal adhesions, but these were not detected in any setting (not shown). The clusters started to disappear 30 min after the beginning of chemokine stimulation in a comparable manner for both C I and FN. (fig. 7)

## Discussion

The interaction of tumour cells with ECM components is an important step in organ-specific metastasis formation. In order to invade potential metastatic target organs, tumour cells are usually required to establish specific adhesion to vascular endothelial cells and/or the organ's ECM. Since metastatic colonization of the liver is one of the most important life-threatening events in breast cancer, we investigated potential mechanisms that might mediate this process. Müller et al. [12] showed that chemokines and their receptors can promote colonization of breast cancer cells into their targets, such as lymph nodes, lung, liver and bone marrow, all of these being sites where chemokine ligands are mainly expressed. For example, Kupffer cells and sinusoidal endothelial cells can secrete CXCL12 that is the ligand for CXCR4 [28]. Breast cancer cells specifically express functionally active CXCR4 and CCR7, which can trigger actin polymerization, pseudopodia formation and directional movements [12]. In this study, we investigated the role of CXCR4/CXCL12 in breast cancer cell migration and adhesion with a focus on its dependence on liver ECM components that may provide an organ-specific microenvironment for rapid tumour cell extravasation into this major metastatic organ.

In our study, breast cancer cells with differing metastatic potential demonstrated different cell adhesion to ECM components in vitro and slightly different migrative properties. Both investigated breast cancer cell lines expressed functionally active CXCR4 at their surfaces and their in vivo potential for chemotactic extravasation rates into the liver parenchyma was comparable. Two clones of MDA-MB-231 cells were transduced with retroviral shRNA [27] to inhibit CXCR4 expression. This resulted in decreased extravasation in vivo, but did not affect cell adhesion within the sinusoids. However, only wild-type controls, but not shRNA-control transductions, were available for this study. Although cell viability was not altered in the transduced clones, some toxic effects cannot be completely ruled out. These intravital results were confirmed by in vitro behaviour in our study and by Chen et al. reporting the inhibition of breast cancer invasion/migration by down-regulation of CXCR4 [18]. In contrast, the adhesive properties of the cells were not influenced by chemokine stimulation, results that are similar to those found by Fernandis et al. [19] for MDA-MD-231 cells. Overexpression of CXCR4 was not used since both cell lines have high percentages of CXCR4-positive cells. In addition, the receptor availability at the cell surfaces is strictly regulated and therefore, overexpression of CXCR4 would be unlikely to result in increased cell surface availability. These findings suggest that CXCL12/CXCR4-mediated chemotactic extravasation of breast cancer cells is required for metastatic colonization of the liver. Since less metastatic cells showed less initial tumour cell arrest within the liver, these initial adhesions - but not subsequent extravasation - appear to be rate-limiting for hepatic metastasis formation.

In our current study, we observed a dose- and matrix-dependent CXCL12-induced migration of breast cancer cells. The bell-shaped response of the migration rates to different concentrations of CXCL12 has also been shown by others [9], [18]. Decreased migration at higher concentrations may be caused by saturation of CXCR4 ligand binding at cell surfaces and rapid internalization. This appears to result in subsequent lack of GTPase activation. Using breast cancer cells in suspension, Holland et al. [21] found that only highly invasive cells were able to form G protein αβγ heterotrimers with CXCR4. Although non-invasive cells expressed similar CXCR4 surface levels, they did not respond to CXCL12 due to lack of GTPase activation. Although MDA-MB-231 and MDA-MB-468 cells had comparable basic in vitro cell motility at all ECM components in our study, their response to CXCL12 stimulation differed between the ECM components. MDA-MB-468 cells mainly responded to CXCL12 stimulation when plated at FN. In contrast, in MDA-MB-231 cells' chemotactic response in the presence of C I and LN were found to be more pronounced than in MDA-MB-468 cells. The slight increase in cell migration under the influence of anti-CXCR4 is likely caused by a partial agonistic effect of this antibody as has been described for many function-blocking antibodies. Since C I appears to be the main “road” for tumour cell extravasation into the liver parenchyma, the enhanced chemotactic motility at C I might be an important determinant for liver colonization. It can be speculated that MDA-MB-231 cells, which were also very motile when LN was present, may use this ECM component to metastasize into target organs where LN is expressed in higher amounts, such as in the lung. This would result in earlier escape from the potentially toxic environment within the microvessels [31].

In cancer cells, different combinations of chemokine receptor activation and integrin binding appear to enable different migration programs, such as mesenchymal-amoeboid transition or collective-amoeboid movement. This form of rescued migration has been shown to be closely related to integrin-ECM interactions [32]. Potential interactions between integrin binding and chemotactic responses were previously reported for different tumour entities. For example, integrin-mediated pancreatic cancer cell migration at LN was found to up-regulate CXCR4 and IL-8 expression and responsiveness to CXCL12 stimulation [33]. In addition, CXCL12 induced redistribution of various integrins between cell surface and intracellular compartments in renal carcinoma cells [34]. In the breast cancer cells investigated in our study, similar alterations of integrin expression during exposure to CXCL12 were not observed.

We found that integrin-mediated cell adhesion at ECM components can specifically induce redistribution of CXCR4 at tumour cell surfaces with formation of larger receptor clusters. Since this was accompanied by intensified formation of lamellipodia and pseudopodia, it is likely that specific integrin-mediated cell adhesion can induce increased CXCR4 sensing [12] and subsequent ability for rapid extravasation along ECM components. These morphological alterations after CXCR4 activation were strong in the highly metastatic MDA-MB-231 cells, and present to a lesser extent in the less metastatic MDA-MB-468 cells. We therefore assume that interactions between integrins and chemokines during chemotactic tumour cell extravasation are more related to outside-in signalling, inducing higher chemotactic cell motility with subsequent faster extravasation. In addition, inside-out activity can improve sensing of chemokine gradients.

A number of cross-signalling pathways between CXCR4 and integrins may be responsible for these ECM-dependent chemokine effects, such as PI3K-AKT [16], [35], FAK-Crk, RAFTK/Pyk2 [19]. Besides kinases, chemokine receptors and integrins can activate or modulate small GTPase signalling pathways [36], [37]. RhoA promotes the contraction and retraction of the cell body at its rear during directed motility. Rac can induce membrane protrusion at the front of the cell and Cdc42 regulates direction of migration by regulating cell polarity [38]. In our study, their CXCL12-induced activation in cell suspensions and increased responsiveness of cells adherent to C I corresponded to the motility response of the cell lines. Limited GTPase activation in cells in suspension without adhesive interactions is likely caused by lost cell polarity that cannot be developed in a proper manner without adhesive contacts. The requirement of specific, integrin-mediated adhesive contacts for sufficient CXCL12 stimulated GTPase activation in our study appears to promote increased chemotactic tumour cell motility in various metastatic target organs. This is further supported by previous observations that GTPase activation can be differently modified by adhesion of various (tumour) cell types to various ECM-components [34], [39], [40].

As previously reported, composition of the ECM within the liver can provide certain structures that may act as guidance for tumour cell extravasation. These migrative properties can be influenced by interactions of integrins with different ECM-components including FN and type IV collagen for initial cell arrest, and C I for extravasation [6], [7], [24]. Sheets of FN and very small amounts of LN within the subendothelial space of Dissé and fibres of C I occurring between hepatocytes could form a path for adherent cells to migrate into the liver parenchyma after sufficient stimulation [24]. Although some differences in the integrin surface expression were found in the breast cancer cells in this study, they cannot solely explain the differing migrative properties. Since tumour cell adhesion but not extravasation correlated with the metastatic potential of the cells, initial arrest within the liver sinusoids appears to be more important as rate-limiting factor in breast cancer metastasis. This is in contrast to other carcinoma entities where rapid extravasation was central to determine metastatic potential [27], [28].

These findings suggest that the combination of chemokine availability at optimal local concentrations, specific ECM composition with differential integrin binding of tumour cells and interaction of chemokine receptors and integrins via GTPase activation are determinants of the extent and time course of tumour cell extravasation into potential metastatic target organs.

### Conclusions

In summary, our results show that chemokines appear to be involved in metastatic tumour cell migration and motility in an organ-specific manner. Using in vitro and intravital observation techniques, we were able to demonstrate that CXCR4 plays an important role in guiding breast cancer cells to target organs, such as liver, due to integrin-adhesion dependent activation, [41]. This finding is further supported by the fact that the required cross-signalling between integrins and chemokine receptors for chemotactic cell motility is ECM-dependent. Availability of chemokine receptors at tumour cell surfaces, presence of their ligands within the microenvironment of potential target organs and the suitability of their ECM composition seem to be required for successful tumour cell extravasation as early steps of metastasis formation.

## Supporting Information

Video S1 **Time-lapse microscopy of MDA-MB-231 at LN in the presence of 25 ng/ml CXCL12 for 60 min.**
(AVI)

Video S2 **Time-lapse microscopy of MDA-MB-468 at LN in the presence of 25 ng/ml CXCL12 for 60 min.**
(AVI)
